# Closing the loop on morphogenesis: a mathematical model of morphogenesis by closed-loop reaction-diffusion

**DOI:** 10.3389/fcell.2023.1087650

**Published:** 2023-08-14

**Authors:** Joel Grodstein, Patrick McMillen, Michael Levin

**Affiliations:** ^1^ Department of Electrical and Computer Engineering, Tufts University, Medford, MA, United States; ^2^ Allen Discovery Center at Tufts University, Medford, MA, United States; ^3^ Wyss Institute for Biologically Inspired Engineering at Harvard University, Boston, MA, United States

**Keywords:** regeneration, development, reaction-diffusion, model, simulation, robust morphogenesis, robust reaction-diffusion

## Abstract

Morphogenesis, the establishment and repair of emergent complex anatomy by groups of cells, is a fascinating and biomedically-relevant problem. One of its most fascinating aspects is that a developing embryo can reliably recover from disturbances, such as splitting into twins. While this reliability implies some type of goal-seeking error minimization over a morphogenic field, there are many gaps with respect to detailed, constructive models of such a process. A common way to achieve reliability is negative feedback, which requires characterizing the existing body shape to create an error signal–but measuring properties of a shape may not be simple. We show how cells communicating in a wave-like pattern could analyze properties of the current body shape. We then describe a closed-loop negative-feedback system for creating reaction-diffusion (RD) patterns with high reliability. Specifically, we use a wave to count the number of peaks in a RD pattern, letting us use a negative-feedback controller to create a pattern with *N* repetitions, where *N* can be altered over a wide range. Furthermore, the individual repetitions of the RD pattern can be easily stretched or shrunk under genetic control to create, e.g., some morphological features larger than others. This work contributes to the exciting effort of understanding design principles of morphological computation, which can be used to understand evolved developmental mechanisms, manipulate them in regenerative-medicine settings, or engineer novel synthetic morphology constructs with desired robust behavior.

## 1 Introduction: reaction/diffusion, positional information and scaling

The generation of complex form during embryonic development, and its repair and remodeling during regeneration, highlight fundamental problems that range from cell and evolutionary biology to control theory and basal cognition (Pezzulo and Levin, 2015; Pezzulo and Levin, 2016; Harris, 2018; Pezzulo, 2020). How can collections of cells cooperate to reliably produce the same species-specific target morphology? Moreover, what mechanisms enable them to robustly do so despite various perturbations? For example, planarian flatworms regenerate their entire body from large or small fragments of any type (Cebrià et al., 2018), while amphibian embryos maintain the right proportions even when many cells are missing (Cooke, 1979; Cooke, 1981) or made too large (Fankhauser, 1945a; Fankhauser, 1945b). This homeostatic property of multicellular morphogenesis has numerous implications beyond basic science, as it represents an attractive target for regenerative medicine and synthetic bioengineering approaches that seek efficient methods for the control of growth and form. A number of mathematical frameworks have been developed to help understand, predict, and control the decision-making of cells in the morphogenetic problem space. Here, we first review several popular approaches to modeling this process, highlighting their positive features and limitations. We then propose a new model which has interesting and useful features for quantitative modeling of morphogenesis.

Negative feedback is a very common and effective means of achieving reliability in nature (Alon, 2019). We will consider negative-feedback systems to arrive at a target body shape. We consider, as an example, the specific question of how morphogenesis creates bodies with five toes rather than four or six. In mice, this has been shown to be accomplished with a five-peaked reaction-diffusion pattern (Raspopovic et al., 2014).

More generally, reaction-diffusion (RD) and positional information (PI) are perhaps the two best known hypotheses in the field of morphogenesis. Green (Green and Sharpe, 2015) gives an excellent summary of both hypotheses as well as contrasting the two. RD (Turing, 1953; Gierer and Meinhardt, 1972; Kondo and Miura, 2010; Marcon and Sharpe, 2012; Meinhardt, 2012; Green and Sharpe, 2015; Painter et al., 2021) was proposed by Turing in 1952. In its simplest form, it uses two chemical species, *A* and *I*. *A* (the “activator”) generates more of *A* and/or *I* via chemical reactions; *I* (the “inhibitor”) similarly reduces their concentrations. Surprisingly, combining these reactions with the diffusion of both *A* and *I* can, in many cases, amplify small random concentration gradients into definite and striking patterns (see (Kondo and Miura, 2010) for many examples).

Intuitively, the activator *A* promotes more of both *A* and *I*. Thus, any small excess of *A* at any location quickly grows by positive feedback. Of course, [*I*] also grows at the same location; but *I* is assumed to diffuse faster than *A*, so there is soon relatively little of *I* at this peak, and so the peak stays a peak. The *I* near the peak prevents new peaks from forming until you get far enough away for [*I*] to drop, at which point the pattern repeats. This concept, local self-activation with long-range inhibition, has been the basis of most RD systems [though new versions have also been discovered (Marcon et al., 2016; Landge et al., 2020)]. All of the variants have the basic ability to start with small, random concentration variations and amplify them into stable large-scale patterns.

Almost 20 years after Turing, Lewis Wolpert published his positional-information hypothesis (Wolpert, 1969). It is attractively simple. First, some unspecified process creates a gradient of a morphogen from, say, head to tail. Next, cells use a gene regulatory network (GRN) to determine their position by sampling the morphogen gradient, and then differentiate accordingly. PI gained rapid popularity. But it never *per se* explained where the initial gradient came from. Furthermore, most morphogen gradients exhibit exponential decay, which implies that much of the field will contain very low concentrations. This would make it difficult (Lander, 2007) for a GRN to determine spatial locations in those areas.

RD is not inherently scalable. RD patterns have a characteristic length λ_RD_, typically given by $\lambda_{RD}=\sqrt{\frac{D_{A}}{K_{D,A}}}$ (where *D*_A_ is the diffusion constant of *A* in m^2^/sec and *K*_D,A_ is the degradation constant of *A* in sec^−1^). In a field of length *L*, an RD pattern typically repeats *L*/λ_RD_ times. Thus, longer fields typically result in more pattern repetitions. This was originally seen as an argument against RD (Marcon and Sharpe, 2012); while it is reasonable for larger animals to have, say, more spots, we would not expect a larger embryo to have extra fingers or toes. This objection was eventually partially overcome. Gierer and Meinhardt first proposed (Gierer and Meinhardt, 1972) a scale-independent version of RD. The advent of modern molecular-biology techniques produced evidence (Raspopovic et al., 2014) that mouse digits are formed with an RD system that uses feed-forward techniques–a molecule that affects embryo size also feeds forwards to affect λ_RD_, thus keeping λ_RD_ reasonably aligned with embryo size and tending to produce the correct number of digits.

**FIGURE 1:**
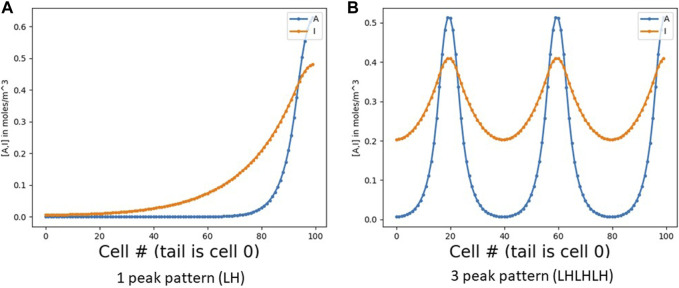
RD pattern profiles in 1 dimension. **(A)** Shows a simple one-peak pattern, otherwise called LH (low [*A*] on the left and high [*A*] on the right). **(B)** Shows a three-peak pattern (LHLHLH). Note that the inhibitor *I* spreads wider than *A* due to its higher diffusion coefficient. In both figures, the *x* axis is cell number (numbered from 0 at the left). With a constant cell diameter *d*, the number of cells is roughly the field length *L* divided by *d* (with some small inter-cell spacing as well).

Barkai later proposed *expansion-repression* (Ben-Zvi and Barkai, 2010; Ben-Zvi et al., 2011), which uses a morphogen *A* and “expander” species *E*. *A* is generated at one end of the field at *x* = 0, and then diffuses and decays everywhere, again with a characteristic length of $\lambda_{RD}=\sqrt{\frac{D_{A}}{K_{D,A}}}.$ Thus [*A*] falls off as *x*>λ_RD_. Because *E* is generated only when [*A*] is less than some repression threshold *T*_rep_, then *E* serves as a way to detect that λ_RD_ is shorter than *L*. They propose that *E* diffuses very quickly and causes λ_RD_ to increase everywhere (either by increasing *D*_A_ or by decreasing *K*_D,A_). By using [*E*] to alter λ_RD_, they robustly set λ_RD_ = *L*/2 and create exactly one repetition of an RD pattern, proportionally scaled to *L* (Figure 1). This is a negative-feedback system, where [*E*] essentially measures *L*/λ_RD_ and then feeds back to adjust λ_RD_.

RD and PI were typically seen as competing theories, until experimental evidence mounted for each being used in different circumstances. Green (Green and Sharpe, 2015) suggested that RD and PI can work together in the same system, e.g., by having RD lay down a gradient that PI then uses; Tewary et al. (2017) is an example of this. So is expansion-repression; it lays down a scalable one-peak pattern, thus creating a morphogen gradient varying from low at one end of an organism to high at the other. Since it is scale independent, the coordinate system scales with the length of the organism.

The central problem is how to take an existing set of features, which may or may not be correct, and move in the correct direction in morphospace. Our understanding of the capability of RD to adapt to the field length *L* has clearly improved over time; from not at all in Turing’s original work (Turing, 1953), to the simple feed-forward hypothesis for mouse digits (Raspopovic et al., 2014), to negative feedback in expansion-repression (Ben-Zvi and Barkai, 2010).

But the negative feedback in expansion-repression only applies to RD patterns with a single peak. How might we get this level of reliability for, say, a five-peak pattern that creates toes? One way could be by adding reliability to RD by wrapping it in a negative-feedback controller. This would require counting the peaks for the current λ_RD_, comparing it to five and adjusting λ_RD_ as needed. But how do we count the number of peaks? While this may seem simple, it is not as easy as it seems; here, we accomplish it using waves.

Why is not it easy? Computation outside of the nervous system is typically done with gene-regulatory networks (GRNs). A GRN is a system of promoters inside a cell that senses messenger signals, turns genes on or off to control protein levels, and in turn creates new messengers. Communication between cells then happens as signals leave cells, and travel by diffusion or gap junctions between neighboring cells. We thus have large numbers of small, independent computing entities, working essentially independently and driven largely by local communication–but unfortunately attempting to solve a global-scale problem.

This type of computation is reminiscent of a cellular automaton (CA). A CA is a large collection of identical processing units (called cells, by analogy to biological cells). Each cell in a CA communicates directly only with its neighbors and indirectly via a chain of cells (analogous to diffusion working most quickly at close range). In fact, our counting problem is similar to the classic majority-detection problem for a CA, where you must look at a field of cells that are either 0 or 1, and determine if there are more 0s or more 1s. While the problem may seem simple, it is not at all so (Gács and Levin, 1978; Fates, 2013).

CAs have been used to navigate morphospace; e.g., by Mordvintsev (Mordvintsev et al., 2020) to robustly create images. However, (Mordvintsev et al., 2020) uses an entire neural network in each cell, which is an unrealistic amount of compute power. Furthermore, like many deep neural networks, theirs is so complex that it is difficult to know if it is correct, let alone understand how to modify it to generate slightly different shapes. CAs have also been used to evolve spiking neural nets for the purpose of evolving artificial brains (de Garis et al., 1993). Most CAs use discrete (e.g., Boolean) state variables, though some CAs use analog state [e.g., the floating-point numbers in (Mordvintsev et al., 2020)].

There are several simulators that are capable of simulating networks of cells with GRNs, as well as reactions and diffusion between the cells. Garmen (Kaul et al., 2023) models GRN nodes as multilevel, with state variables either on, off or medium. PhysiBoss (Letort et al., 2019) is a combination of MaBoSS for Boolean models of the GRN and multicellular behaviour using agent-based modelling (PhysiCell). CompuCell 3D (Swat et al., 2012) is a well-known simulator based on the Cellular Potts model. It can simulate a wide variety of partial differential equations (PDEs). Any of the above simulators would probably have been suitable for this work. We use a simulator based on BETSE (Pietak and Levin, 2016; Pietak and Levin, 2017), largely for our convenience based on familiarity with it. Our simulator supports numerical integration of the PDEs for diffusion and for chemical reactions by efficient implicit techniques.

Waves of various types have been well researched in biological tissues (Deneke and Di Talia, 2018; Durston et al., 2018), regulating outcomes ranging across cell death (Cheng and Ferrell, 2018), proliferation (Anderson et al., 2017), and handedness (Anderson et al., 2017). Differentiation waves (Gordon and Gordon, 2019; Gordon and Stone, 2021) are mechanical waves that have been proposed as the driving force behind embryonic differentiation. It was hypothesized that a mechanical differentiation wave reaches a cell, which then decides (via a “cell-splitter” organelle) between one of two resultant states, and which results in one of two refinements of the cell state as well as potentially launching a new differentiation wave. The computational methods by which a differentiation wave might cause a state decision to be reached are unknown. By contrast, our wave is chemical rather than mechanical, and we are proposing a specific GRN to, specifically, count.

Chhabra et al. (2019) describes a fascinating example of waves during gastrulation of human embryonic stem cells *in vitro*. After seeding with BMP4, they see waves of WNT and NODAL signaling, with cell differentiation happening along with the waves. They show that the cell fates are not decided as a result of reading static concentration gradients left by RD. Rather, the waves are not part of a RD process but arise from some other method; and the cells decide their fate dynamically during the waves. In fact, the WNT and NODAL waves eventually die and concentrations become homogeneous, but the cell fates remain. They have thus used waves as an integral part of differentiating a gastrula into its three layers. By contrast, we will divide a tissue into an arbitrary number of layers, and easily make some layers larger or smaller, using waves that are part of a robust negative-feedback process.

**FIGURE 2:**
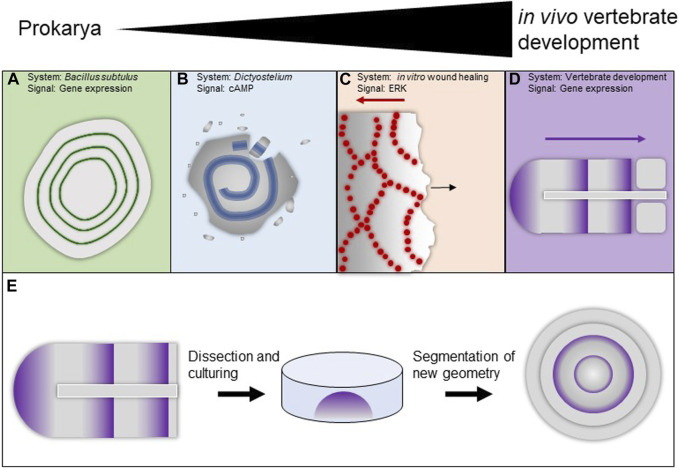
Examples of waves in biological systems **(A–E)**. **(A)** Gene expression waves in bacterial biofilms. **(B)** Circular cAMP waves in *Dictyostelium* facultatively social amoeba. **(C)** Waves of ERK signaling in MDCK cells in response to wounding. **(D)** Gene expression waves during vertebrate segmentation. **(E)** Cultures paraxial mesoderm tissue will form segments in its new variant geometry.

Waves have been observed in many different biological systems using disparate signaling modalities. *Bacillus subtulus* biofilms produce systemic oscillations in response to nitrogen stress [Figure 2A (Chou et al., 2022)]. Colonies of facultative social Dictyostelium amoebae produce chemoattractive spiral waves of cAMP [Figure 2B (Fujimori et al., 2019; Singer et al., 2019; Ford et al., 2023)]. In cultured mammalian MDCK cells, Erk signaling waves manifest in response to wounding [Figure2C (Aoki et al., 2017; Hino et al., 2020)]. The *Xenopus laevis* embryo displays numerous waves from the fertilization-triggered calcium wave that initiates development (Busa and Nuccitelli, 1985) to the cell cycle trigger waves that coordinate early cell divisions (Chang and Ferrell, 2013; Anderson et al., 2017), to travelling gene expression waves that help establish the segmental pattern of the embryo (Jen et al., 1999).

Systems-level wave phenomena are likely under-reported in biological datasets as their detection requires long-term live imaging with faithful dynamic reporters. Many commonly used endpoint techniques, like *in situ* hybridization and immunohistochemistry, are poorly suited to detect dynamic patterns as they require the tissue to be fixed. Others, like RNA sequencing, remove both temporal and spatial information from the assayed tissue. Recent breakthroughs in live imaging of non-neural tissue signaling have revealed a diverse array of mesoscale temporospatial dynamic patterns.

Perhaps the most studied example of waves of gene expression is the segmentation clock, which helps establish the segmental pattern of the vertebrate axis [Figure 2D (Palmeirim et al., 1997; Soroldoni et al., 2014)]. It originated as the clock-and-wavefront model (Cooke and Zeeman, 1976; Tabin and Johnson, 2001) and typically requires a global clock; some versions (Cotterell et al., 2015) avoid this requirement and have an RD-like flavor. It is able, across species, to produce a variable number of vertebrae.

Furthermore, the segmentation clock is remarkably resistant to perturbation. Dividing cells add noise to the system, lose their phase but are re-synchronized to the phase of their neighbors via local interactions (Zhang et al., 2008). When segmentation is transiently chemically interrupted it will reliably recover, even when clock components are heterozygously mutated (Mara et al., 2007). When paraxial mesoderm is surgically removed and cultured *ex vivo* it will manifest waves and form segments in its new geometry [Figure 2E (Lauschke et al., 2013)], and even when completely dissociated and re-aggregated it will re-establish coordinated oscillations (Tsiairis and Aulehla, 2016; Hubaud et al., 2017).

Despite decades of research, many details of the segmentation clock–including reasons for its robustness–remain unknown. Some segmentation-clock errors do not seem to self-correct (Pourquie, 2022). Its waves are an integral part of the differentiation process. By contrast, our waves perform measurement, are part of an even more robust negative feedback system, and do not require a clock.

Our work, then, combines the two themes of robust morphogenesis and wavefronts. We introduce a GRN that, when replicated in cells and properly stimulated at one end, launches a wave. We will show that the wavefront counts the peaks in an RD pattern as it passes them. This then allows for a closed-loop negative-feedback system to robustly control the number of peaks. In our system, observable waves of morphogen concentration essentially occur as a side effect of computing the properties of a tissue.

Consider a simple RD pattern on a 1D field of cells. The number of replications of the basic RD pattern will depend on the length *L* of the field and the pattern’s intrinsic length λ_RD_; we typically get roughly *L*/λ_RD_ pattern repetitions in a field of length *L*. Expansion-repression, as noted above, can alter λ_RD_ so that we always get exactly one peak at the source, independent of *L*. We go a step further: given an integer goal *N*, we will alter λ_RD_ so as to instead obtain exactly *N* peaks (Figure 1), again independent of *L*. To do this, we use a wave, based on an identical simple GRN in each cell, that serves to count the number of peaks. We then wrap the GRNs in a top-level control loop that iteratively adjusts λ_RD_ and launches a new wave until the wave counts exactly *N* peaks. Essentially, we have built a closed-loop negative-feedback goal-seeking machine for morphogenesis. It knows its target pattern shape and adjusts parameters iteratively until that goal is achieved.

**FIGURE 3:**
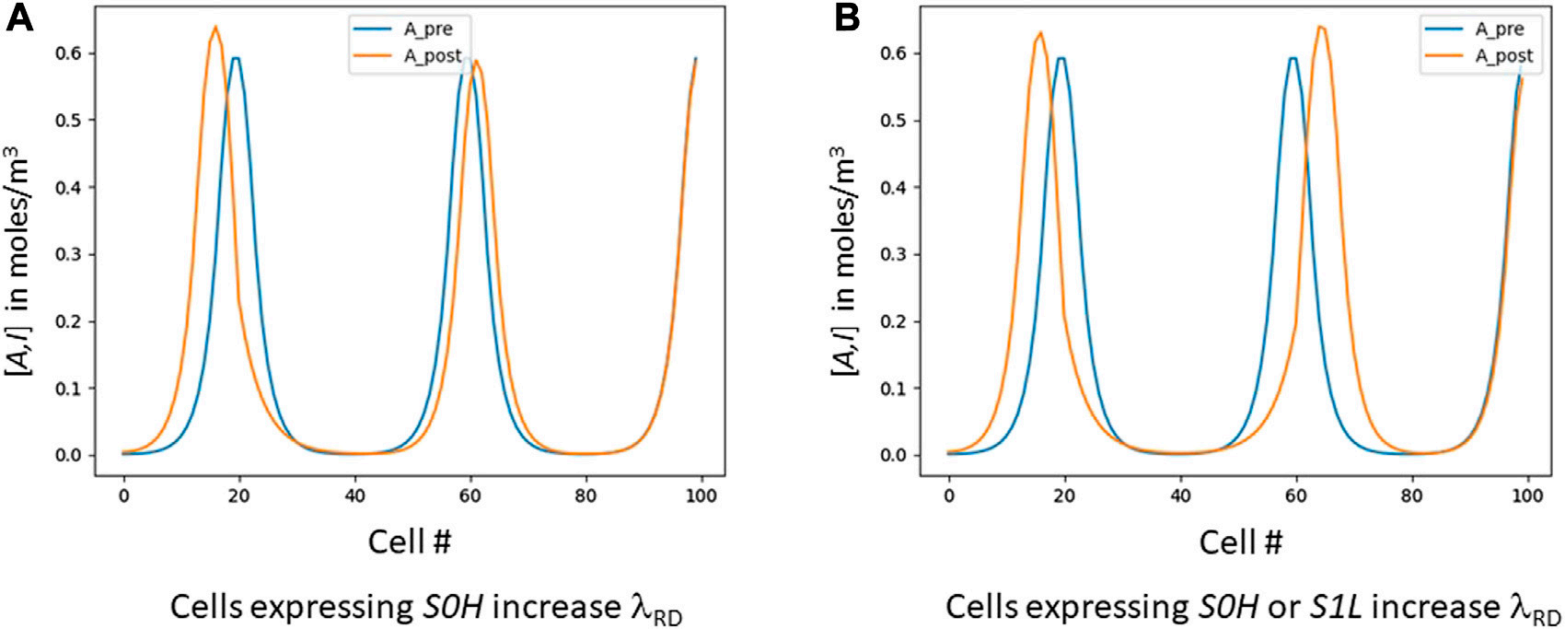
Skewing λ_RD_ digitally. These graphs are the result of a post-process that uses the “bread-crumb” signals S* created by the cellular automaton. The blue lines are the evenly-spaced signals before skewing; the orange shows the results after intentional skewing. **(A)** shows an experiment where all cells that express *S0H* (roughly, cells 18–27) increase their λ_RD_, resulting in that area widening and pushing itself away from the area to its right. **(B)** shows all cells expressing either *S0H* or *S1L* increasing λ_RD_, resulting in the entire first dip (roughly cells 15–65) widening.

We add one more capability. Green (Green and Sharpe, 2015) has proposed systems where RD acts downstream of PI, with a morphogen gradient inducing a gradual increase in λ_RD_ so that, e.g., digits in a mouse paw are wider at their distal end than their proximal end (Sheth et al., 2012). Meinhardt (2021) has proposed a similar mechanism in Hydra. Both of these serve to build an RD pattern where λ_RD_ varies from a small, tight pattern at one end of the field to a larger λ_RD_ at the other end. As our wave counts the peaks in an RD pattern, it leaves behind digital breadcrumbs such that each cell knows its exact ordinal position. A small amount of per-cell logic can then examine those signals and increase or decrease λ_RD_ in any given cell(s). As a result (Figure 3), we too can make λ_RD_ larger or smaller at different locations in the field; but we can do it arbitrarily, rather than only a simple monotonic increase from one end to the other as in (Meinhardt, 2012).

## 2 Methods

### 2.1 Why the GRN is hard

In order to have closed-loop negative feedback to create a RD pattern with the desired number of peaks, we must be able to count the current number of peaks. That is what our GRN does–but unfortunately, counting is more difficult than it may seem. To demonstrate why, we’ll start with a “strawman” solution whose failings will motivate the actual solution.

A human six-year-old, looking at the patterns in Figure 1, can easily tell that Figure 1A has one peak and Figure 1B has three. He would basically scan the picture from left to right, counting each peak as it occurs. But how can an organism, using only a simple GRN replicated in every cell, perform this task? We might start with a set of signals *S0*, *S1*, *S2*, etc., denoting the number of peaks to any cell’s left.

Consider a strawman GRN in each cell that implements logic such as

rising_edge = ([*A*]_left_<.2) and([*A*]_me_>.2)

if (I am a rising-edge cell)

pump out the next higher signal than I see on my left

**FIGURE 4:**
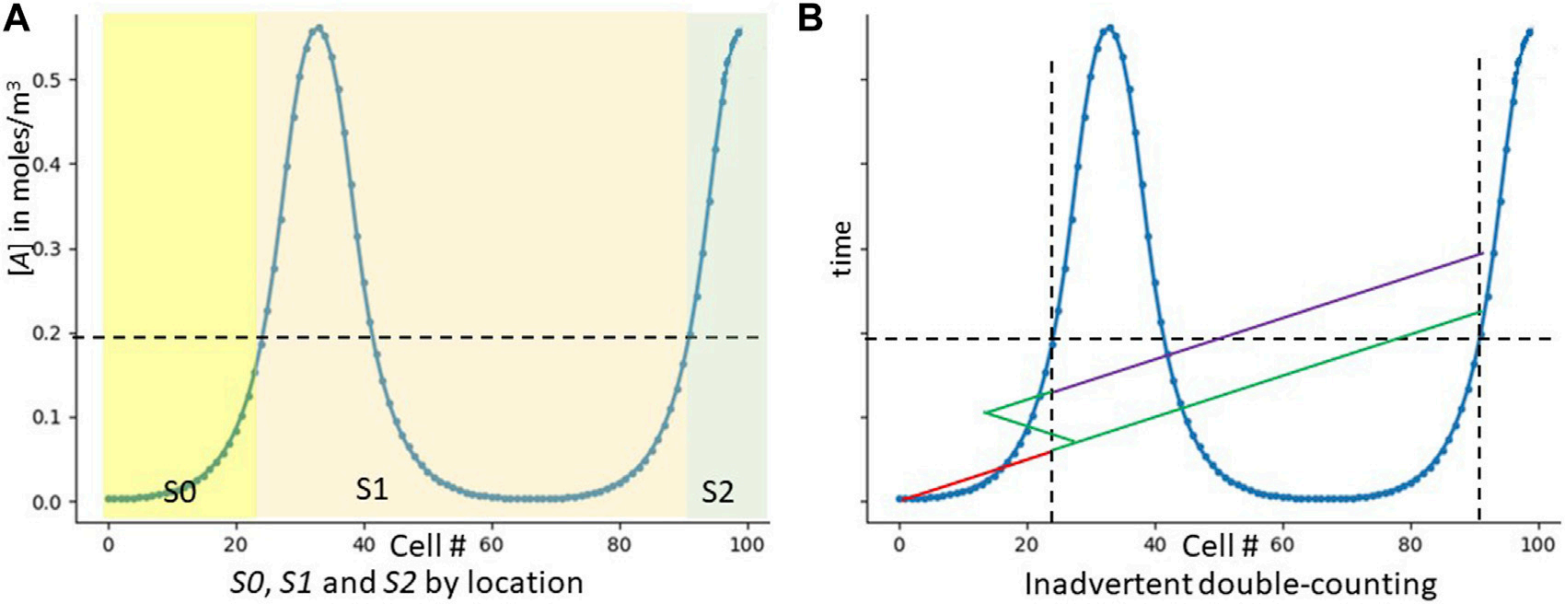
The strawman GRN. The “strawman” GRN does not work, but shows why counting peaks is hard. **(A)** Shows which cells express which signals in our original strawman GRN. **(B)** Shows inadvertent double counting. The *y* axis is now time, not concentration. The red line shows the signaling of *S0*. As it reaches cell 23, that cell notes the threshold crossing and emits *S1* (green). *S1* not only diffuses correctly to the right, but inadvertently to the left. As it is regenerated from these leftward cells, they also re-emit it, and it travels to the right. When it then crosses cell 23, the cell now incorrectly emits *S2* (purple). Only one inadvertent path is shown–many more are possible.

We are assuming that, for any given cell, [*A*]_me_ is its own concentration of the RD activator *A*, and [*A*]_left_ is the concentration of *A* in the cell on its left. And while we have expressed this logic in terms of Boolean AND gates and IF statements, it can easily be translated into a GRN (Alon, 2019). Figure 4A shows which signals should be expressed in which locations as per this strawman scheme.

However, multiple issues prevent the strawman GRN from actually producing Figure 4A. The first issue is directionality and loops. Our six-year-old human knows to count by sweeping his vision *unidirectionally* from left to right. Cells have no inherent concept of left and right; and signaling molecules, traveling by diffusion, move in all directions equally well. We would thus be susceptible to improper counting with a scenario like Figure 4B. How can we implement unidirectional counting when our signalling molecules diffuse equally well in all directions? Issues like this make CA algorithms challenging (Gács and Levin, 1978; Fates, 2013).

**FIGURE 5:**
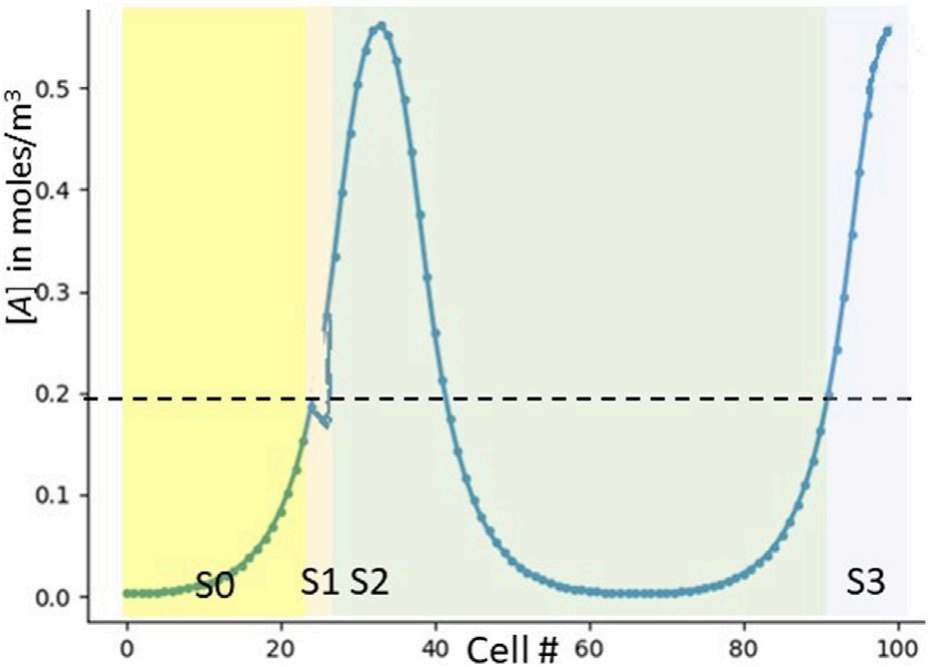
Noisy signals causing double-counting in the strawman GRN. We have drawn a small downwards noise blip right about the cell where [*A*] = .2. This blip cause two cells–first the correct one and next the inadvertent one–to notice a transition rising past [*A*] = .2. The final result is an extra count (detecting three peaks rather than two).

But the problems with this strawman solution are not over. We’ve talked about a cell measuring [*A*]_left_ without explaining how that communication could happen. And even if it could happen, our system thus far is not robust to noisy signals. For example, if [*A*] had some noise that caused wiggling around the cell where [*A*] = .2, our GRN might miscount the noise as an extra peak (Figure 5). Biology is noisy (McAdams and Arkin, 1999), and real-life systems must be robust.

### 2.2 The effective GRN

We can resolve our second and third issues with a simple trick that is very common in human-designed noise-filtering schemes, called a *Schmidt trigger* (Holdsworth and Woods, 2002). A Schmidt trigger does not try to detect edges directly, but instead uses hysteresis. Instead of trying to detect an edge at 0.2, it uses *two* thresholds; e.g., .1 and .3. When the signal falls below .1, it is noted as low; when it rises above .3, it is noted as high; and an edge is counted when we rise from low to high. Any spurious noise between .1 and .3 has been made irrelevant.

What governs the choice of, in our case, .1 and .3 as our new thresholds? The further apart the two thresholds are, the more noise immunity we have. On the other hand, if we make (e.g.,) the .3 too high, we risk having some peaks that never reach that threshold and are not counted.

**FIGURE 6:**
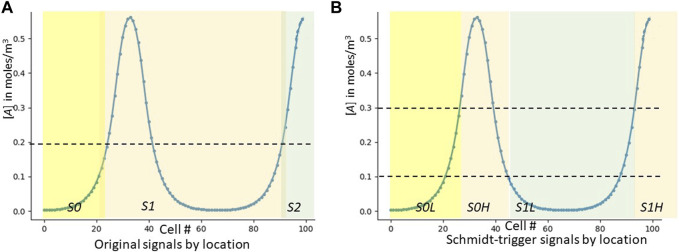
Which signals are expressed by which cells in the updated GRN. As an aid to understanding how the cellular automaton works, we graphically show which cells express each of the S* signals. **(A)** is for the original strawman algorithm (a duplicate of Figure 3A). **(B)** is for the improved algorithm using Schmidt triggers.

We have now fixed our robustness-to-noise issue, and in fact also no longer need a cell to measure any concentration other than its own. We have incurred the cost of now needing two signals to count each peak: our new signals *S0L*, *S0H*, *S1L*, *S1H*, etc., are now generated as per Figure 6.

We must still deal with the problem of directionality, loops and double counting (Figure 4B). Our trick is to take advantage of having a prior process break symmetry and give us a known head and tail, thus enabling a global unidirectional sweep to simply count. In this sense, our model is a contribution to the classic problem of leveraging large-scale morphogenetic order from molecular symmetry breaking (Brown and Wolpert, 1990; Vandenberg and Levin, 2009; Vandenberg and Levin, 2010; Naganathan et al., 2016).

We implement unidirectional signaling by tying computation to a wavefront. Cell #0 (at the left) initiates the wave by generating *S0L*. When cell #1 sees the wavefront, it looks at the local [*A*] and decides whether to regenerate *S0L* or to instead generate *S0H*. Importantly, it then freezes its decision until the next wavefront (if any) happens. It finally sources the appropriate signaling molecule (*S0L* or *S0H*) that it wants to travel to cell #2. Of course, these molecules travel to the left towards cell #0 as well–but it is moot, since all cells on the left have already seen the wavefront, made their decision, and will not change it until another wave comes.

With this system, even though our signaling molecules diffuse equally in all directions, the computation wavefront proceeds only from left to right. The key is that any path from cell #0 to a cell *C* via a loop will be longer than the simple direct path from cell #0 to *C*, and thus will arrive at *C after* the direct signal has arrived, and thus cannot affect the action that cell *C* takes. As the wave hits any cell, that cell examines the local environment (specifically, [*A*]) and the accumulated state arriving in the wave (i.e., whether the wave is sending *S0L, S0H,* etc.) to decide what signal to send out in the exiting wave to the next cell to the right.

Here is the final robust GRN in each cell (where the top-level controller now seeds cell #0 with S0L):

$$Pre0L=Pre0L\;\left|\;nothing\;\right|\;\left(S0L\;\&\;!very\_high\&\;!done\right)$$

$$Pre0H=Pre0H\;\left|\;\left(S0L\;\&\;very\_high\;\&\;!done\right)\;\right|\;\left(S0H\&\;!very\_low\&\;!done\right)$$

$$Pre1L=Pre1L\;\left|\;\left(S0H\&\;very\_low\;\&\;!done\right)\;\right|\;\left(S1L\;\&\;!very\_high\&\;!done\right)$$

$$Pre1H=Pre1H\;\left|\;\left(S1L\;\&\;very\_high\;\&\;!done\right)\;\right|\;\left(S1H\&\;!very\_low\;\&\;!done\right)$$

…

$$done=Pre0L\;|\;Pre0H\;\left|\;Pre1H\;\right|\;Pre1L\;\left|\;Pre2H\;\right|\;\ldots$$

S0L=Pre0L

S0H=Pre0H

S1L=Pre1L

S1H=Pre1H

The combination of Schmidt triggers with computation wavefronts counts peaks quite robustly; we will discuss the limits of robustness shortly. Again, while we have expressed this logic in terms of simple Boolean AND, OR and NOT gates, it can easily be translated into a GRN (Alon, 2019), where each signal in the GRN becomes either a protein or a messenger induced by a protein. Finally, assume a top-level controller forcing the leftmost cell to express *S0_L*.

Cells communicate with each other with the S* signals. Any cell participates in the computation wave by waiting to receive an S* signal, then deciding (based on the local [*A*]) which S* signal to relay onwards. The Pre* and *done* signals are local (i.e., do not leave the cell that generates them) and assure that each cell computes only at the wavefront. Any cell, once it receives an S* signal, makes its decision by driving one of the Pre* signals. These signals stay within the cell and are purely for internal calculation. Once a cell drives any of its Pre* signals high, its *done* (which also remains in the cell) also goes high. This then feeds back to the first set of equations and serves to cut off the Pre* signals from looking at any incoming S* signal any longer. At this point, the self-loop in each Pre* equation takes over, so that whichever Pre* signal is asserted will stay asserted.

Finally, the appropriate S* signal gets driven out of the cell, and stays asserted until *new_wave* comes in and breaks the Pre* self-loops. *New_wave* is a global (i.e., widely-diffusing) signal that operates by substantially increasing the degradation rate of the Pre* signals (e.g., by adding a degradation tag). This breaks the self-loop, and thus turns off the Pre* and then *done* signals in each cell. Morphogenesis is a dynamic process; cells respond to cues throughout morphogenesis, and there will thus be frequent computation waves. Each is preceded by a *new_wave* signal, which is issued by the top-level controller; while *new_wave* must reach a high enough level everywhere to act as a global signal, it does not need to reach a level of full spatial homogeneity.

Given that each S* signal is merely a buffer of its corresponding Pre* signal, why bother with the extra signals? The self-loop on each Pre* signal implements our memory of the decision a cell takes; if we tried to put that self-loop on the S* signal instead, then each cell would latch the incoming S* signal before making a decision of its own.

In our GRN, the signals Pre*, *done*, *very_high* and *very_low* are proteins, expressed by genes given the appropriate transcription factors. However, the S* signals must travel between cells and are thus unlikely to be proteins and are thus not the direct output of genes; they may, e.g., be created from the gene products Pre* by chemical reactions.

### 2.3 The top-level controller

**FIGURE 7:**
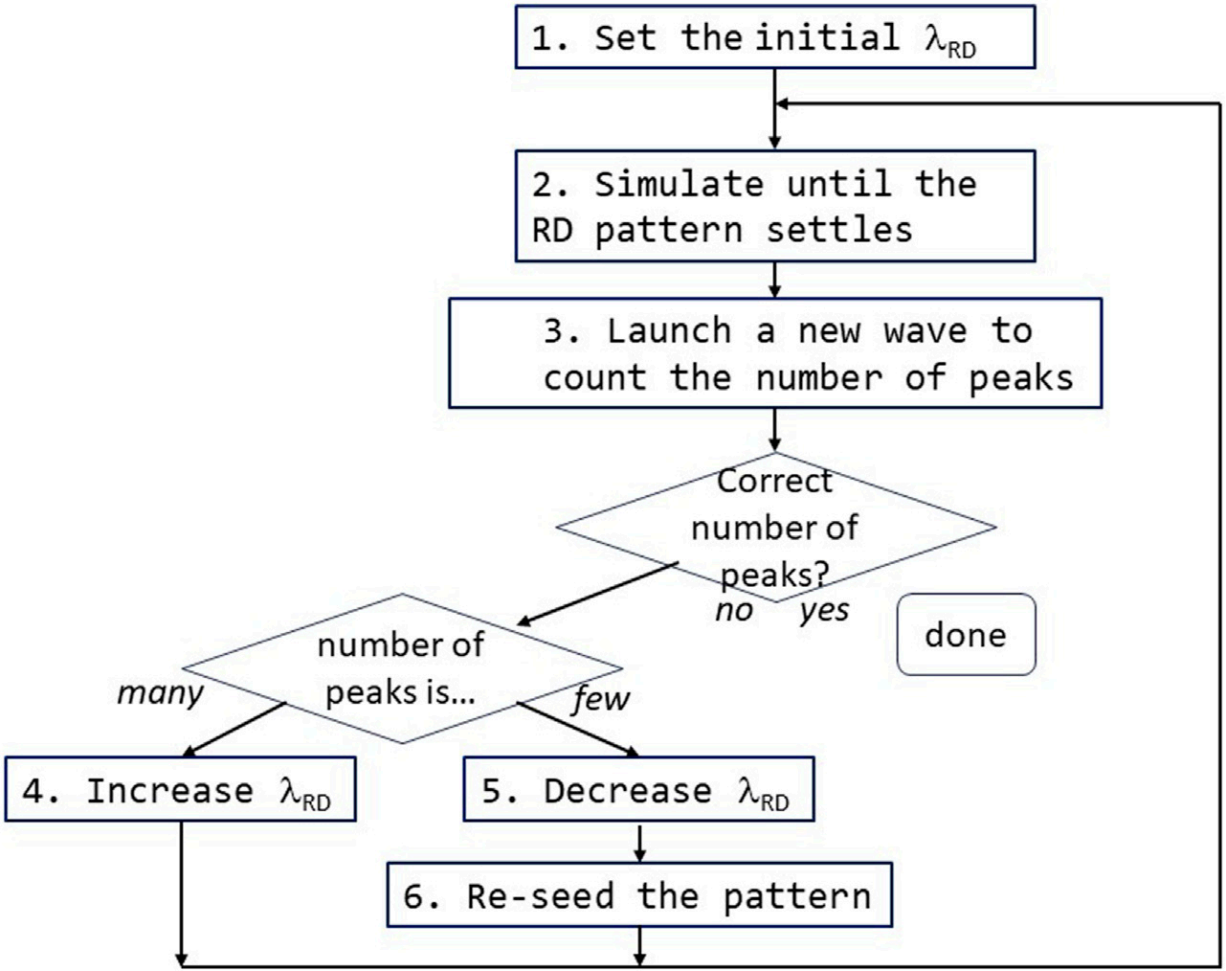
Flowchart of the top-level controller. This figure illustrates how the top-level controller operates.

The next piece in our system is the top-level controller. The controller takes a goal *N*. It uses the computation wave to count peaks; then compares the count to *N* and adjusts λ_RD_ as needed; and iterates this sequence until we have exactly *N* peaks. It is conceptually quite simple (Figure 7). The parenthesized numbers below correspond to the flowchart steps in Figure 7.

The system starts with an initial λ_RD_ (1) and settles to the resulting RD pattern. This is done with an initial simulation run (2). At that point, the controller launches a new computation wave to count the number of peaks in the RD pattern (3). If, fortuitously, we have exactly the desired number of peaks, then we are done. If not, then the controller implements negative-feedback control. If there are more peaks than our goal, the controller will slightly increase λ_RD_ (4). If, on the other hand, there are too few, it decreases λ_RD_ (5) and re-seeds (6). In either case, it then re-simulates (2), and the loop iterates until we have attained our goal.

**FIGURE 8:**
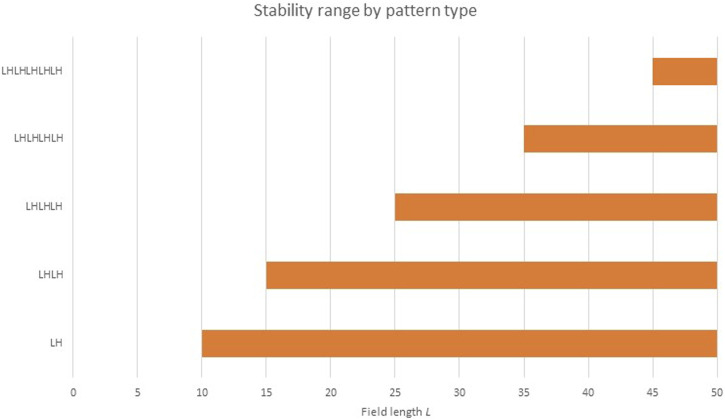
Pattern viability at various *L* values. This figure illustrates how most field lengths *L* can support more than one RD pattern. The LH pattern is viable at all field lengths larger than λ_RD_. At field lengths *L* in the range (Kondo and Miura, 2010; Marcon et al., 2016), both LH and LHLH are viable. At longer L, still more shapes are viable.

What does the “re-seed the pattern” step mean? It has been known since the 1970s (Bard and Lauder, 1974) that the number of repetitions of a Turing pattern is sensitive to initial conditions. Werner has noted (Werner et al., 2015) that while *L*/λ_RD_ is an upper bound for the number pattern replications in a field of length *L*, the lower bound can sometimes be 1 (Figure 8). In other words, while we cannot fit (e.g.,) four RD pattern repetitions in a space only large enough for three, it is possible [albeit unlikely (Werner et al., 2015)] for one single pattern repetition to stretch/scale itself up to an almost arbitrarily large field.

The top-level code above thus includes a small trick. When we are *increasing* λ_RD_, we merely continue the simulation with the larger value. But when we decrease λ_RD_, this may not succeed at creating extra peaks. Instead, it turns out that setting [*I*] = 0, with [*A*] rising linearly from 0 at tail to 1 at the head is reasonably reliable at seeding the maximum number of peaks in a given field size. As discussed below, it is not 100% reliable–but our closed-loop controller can successfully work around the unreliable building block.

The top-level controller must be located where it can see the result of the computation wave; for a tail-to-head wave it must live near the head. The controller is small and simple enough that it could easily be implemented as a GRN. There must be only one top-level controller; if it is located in a cell, then there must be a mechanism for it to only be active in one location. For simplicity, we have merely left it as software in our simulations.

### 2.4 GRN details and limits of robustness

Here, again, is the detailed GRN that implements our cellular automaton:

$$Pre0L=Pre0L\;\left|\;nothing\;\right|\;\left(S0L\;\&\;!very\_high\&\;!done\right)$$

$$Pre0H=Pre0H\;\left|\;\left(S0L\;\&\;very\_high\;\&\;!done\right)\;\right|\;\left(S0H\&\;!very\_low\&\;!done\right)$$

$$Pre1L=Pre1L\;\left|\;\left(S0H\&\;very\_low\;\&\;!done\right)\;\right|\;\left(S1L\;\&\;!very\_high\&\;!done\right)$$

$$Pre1H=Pre1H\;\left|\;\left(S1L\;\&\;very\_high\;\&\;!done\right)\;\right|\;\left(S1H\&\;!very\_low\;\&\;!done\right)$$

…

$$done=Pre0L\;|\;Pre0H\;\left|\;Pre1H\;\right|\;Pre1L\;\left|\;Pre2H\;\right|\;\ldots$$

S0L=Pre0L

S0H=Pre0H

S1L=Pre1L

S1H=Pre1H

We implement the logic equations, as is often done, as Hill functions. The Pre* gates are the most complex: e.g., for *Pre0H* (Eq. 1):

$$Pre0H=k_{v}\frac{{\left(\frac{Pre0H}{.5}\right)}^{3}}{1+{\left(\frac{Pre0H}{.5}\right)}^{3}}+\frac{{\left(S0L/.3\right)}^{3}}{1+{\left(S0L/.3\right)}^{3}}∙\frac{{\left(A/.3\right)}^{3}}{1+{\left(A/.3\right)}^{3}}∙\frac{1}{1+{\left(done/.5\right)}^{3}}+\frac{{\left(S0H/.3\right)}^{3}}{1+{\left(S0H/.3\right)}^{3}}∙\frac{{\left(A/.1\right)}^{3}}{1+{\left(A/.1\right)}^{3}}∙\frac{1}{1+{\left(done/.5\right)}^{3}}$$ (1)

The *done* gates are implemented as $done=k_{v}\frac{{(\frac{\sum Pre*}{.5})}^{3}}{1+{(\frac{\sum Pre*}{.5})}^{3}}.$

Finally, the S* gates are (e.g., for *S0H*) $S0H=k_{v}\frac{{(\frac{Pre0H}{.5})}^{3}}{1+{(\frac{Pre0H}{.5})}^{3}}.$

Robust operation of the computation wave places some constraints on system parameters. For example, the S* signals are meant to travel by diffusion to their nearest neighbors. A molecule *S* generated at a constant rate *G*_S_ moles/s at *x*_0_ and diffusing freely will have its concentration given by $G\left(x\right)=\frac{G_{S}}{K_{D,S}}e^{-\lambda_{S}\left|x-x_{0}\right|}$ , where *K*_D,S_ is the decay rate for *S*, $\lambda_{S}=\sqrt{\frac{D_{S}}{K_{D,S}}}$ and *D*_S_ is the diffusion rate for *S*. Clearly λ_S_ must be large enough for the signal to reach its nearest-neighbor cells, which is hopefully easy. However, it must also be small enough that the S* signals do not travel further than one half cycle of the pattern. So, e.g., *S1H* will first be generated at the cell *C*_0_ where *S1L* is seen and [*A*]>.3; it will correctly be generated at cells further to the right until we reach a cell *C*_1_ where [*A*]<.1. At that point, *S2L* will correctly be generated, and regenerated at successive cells until we reach a cell *C*_2_ where [*A*]>.3 again. But what if *S1H* (traveling by diffusion from cell *C*_1_) reaches cell *C*_2_ before *S2L* (being regenerated at each cell from *C*_1_ to *C*_2_) does? In that case, cell *C*_2_ will incorrectly see *S1H* as the incoming wavefront, and will express *Pre1H* immediately, and will be locked into that decision before it sees *S2L* and tries to express *S2H*. As a result, the count will be too low by one.

How do we avoid this issue? Diffusion is an order (distance squared) process and thus slow over long distances. Our trick then is simply to be sure that the half-pattern-length distance is always more than just one or two cells. This imposes a minimum size on λ_RD_.

### 2.5 Simulation algorithm

In most of the RD literature, the reaction and diffusion can occur anywhere in a homogeneous fixed-length field. Simulating cells that interact with RD then requires interfacing this continuous RD field with cells that are at discretized locations. Often (e.g., the sims in (Kaul et al., 2023) that explain the results from (Tewary et al., 2017; Chhabra et al., 2019)), this is done by stepwise alternation between two simulators; first simulate RD, then each cell reads the concentrations at its discrete location to give to its GRN simulation. Since it is uncommon for RD systems to allow an analytic solution, numerical simulation is used for RD, which discretizes the simulation area in any case. The RD activator *A* and inhibitor *I* might then be small molecules that could diffuse through a cell membrane and then interact anywhere in the field.

Our simulations are done with BITSEY, a faster but less powerful version of BETSE (Pietak and Levin, 2016; Pietak and Levin, 2017). All of the code to reproduce the simulations is publically available at (repository location to be added after the blind-review process https://gitlab.com/grodstein/bitsey/-/tree/master/RD). BITSEY treats the world as a collection of cells interconnected by gap junctions (GJs). Since the proportion of cell surface area covered by GJs is typically small, reactions in a cell tend to happen on a faster scale than inter-cell communication (Pietak and Levin, 2017): BITSEY thus models molecular concentrations as being constant within a cell rather than subdividing a single cell into multiple finite volumes, leading to fast simulation. While BITSEY models ion channels via the Goldman-Hodgkins-Katz (Keener and Sneyd, 2009) model, our current work does not use them. Communication between cells through GJs is modeled by a simple diffusive flow, discretizing Ficke’s Law.

It is easy to show that, as long as the RD pattern length is substantially longer than the diameter of a cell, BITSEY’s model is mathematically equivalent to a simple discretization of the diffusion equations in 1D, where BITSEY essentially chooses the discretization length of numerical integration to be the cell diameter. This, of course, may not be numerically optimal; if the RD pattern size is far larger than a cell, then BITSEY’s model is unduly compute expensive, and if the RD pattern size is on the order of the cell diameter then BITSEY’s model is numerically inaccurate. However, the model does give us one feature–the exact same simulator core handles both RD simulation and GRN simulation, and both could happen simultaneously if desired.

Each cell holds one GRN. Rather than modeling the GRN in each cell by Boolean or multi-level logic [as, e.g., (Kaul et al., 2023)], BITSEY models the differential equations as per a Hill model (Alon, 2019). Thus, the discretization in space is a single cell; the discretization in time is determined by the speed of change in cell-concentration changes, both from the GRNs and from GJs.

There are countless varieties of RD equations in the literature. Most would work equally well in our system, but we had to choose one. We used a very simple RD pattern taken from (Werner et al., 2015), where (Eq. 2)

$$\frac{\partial A}{\partial t}=g_{A}G\left(A,I\right)-k_{D,A}A+D_{A}\frac{\partial^{2}A}{\partial x^{2}},\frac{\partial I}{\partial t}=g_{I}G\left(A,I\right)-k_{D,I}I+D_{I}\frac{\partial^{2}I}{\partial x^{2}}\;and\;G\left(A,I\right)=\frac{1}{1+{\left(I/A\right)}^{h}}$$ (2)

There are numerous more-complex RD systems in the literature, including entire categories of new systems that do not place stringent requirements on diffusivity ratios (Marcon and Sharpe, 2012; Landge et al., 2020). The choice of RD system is immaterial to this work, as long as it can be manipulated by λ_RD_ and it forms an oscillating pattern of some species that the per-cell GRN can see.

The feedback mechanism in our system operates by repeatedly adjusting λ_RD_. Since $\lambda_{RD}=\sqrt{\frac{D_{A}}{k_{D,A}}}$ (where *D*_A_ is the diffusion constant of *A* in m^2^/sec and *k*_D,A_ is the degradation constant of *A* in sec^−1^), this can be done by either adjusting *D*_A_ or *k*_D,A_. Nature has access to multiple means of controlling both of these (Lander, 2007): degradation tags, competing reactions to bind a morphogen, and even lipid modification to affect diffusivity. We choose to alter *D*_A_ by changing GJ density. Altering the density of GJs between cells changes the effective diffusion rate of molecules passing through those GJs. While this choice is largely for convenience, there is evidence that such a mechanism does exist (Kleber and Jin, 2021; Tripathi, 2021). Furthermore, there is substantial evidence (Iovine et al., 2005; Watanabe et al., 2006; Watanabe and Kondo, 2012) in fish models of RD molecules traveling through GJs, so this seemed to be a reasonable choice. Once more, we choose to use this mechanism in our simulations for compatibility with an existing simulator, and it is not central to our results.

Our simulation methodology imposes limits on the values of *N* (the target number of RD pattern repetitions) and *L* (the field length). As *N* gets larger, we are asking for each RD pattern repetition to occur across a smaller number of cells. At some point, quantization errors make the RD patterns less stable. This tends only to be a problem for simulations that (e.g., in order to improve simulation time) use a biologically unrealistically-small field length.

The top-level controller, as noted, is implemented as Python code for convenience. It simply iterates between calling the main simulation engine to let the RD pattern stabilize, simulating again to kick off a new computation wave that counts pattern peaks, and adjusting λ_RD_ accordingly. The simulation engine treats the RD simulation no differently than the GRN simulation and in fact simulates both concurrently. The simulation run that simulates the RD pattern to steady state is also simulating the GRN–but since the top-level controller has not seeded cell #0 with *S0L*, the GRNs never see a wave and are inactive.

### 2.6 Symbols

For convenience, in this section we recap all symbols that have been used globally throughout the paper. We do not include symbols that are used only once and defined at their point of use.

• λ_RD_: the “intrinsic wavelength” of a RD pattern. When the pattern repeats itself, each repetition tends to have length λ_RD_ (and cannot be longer than that).
• *L*: the length of the 1D field that patterning occurs in.
• *N*: the target number of repetitions of an RD pattern. E.g., for the fingers of a human hand, *N* = 5.
• *A*, *I*: the RD activator and inhibitor
• *D*_A_: the diffusion constant (in meters^2^/second) of the activator
• *k*_D,A_: the degradation constant (in 1/second) of the activator
• [*A*]_me_: the concentration of *A* in a cell (i.e., “its own” concentration).
• [*A*]_left_: ditto, but in the cell to the left of the “me” cell. This is only used in the purposely-incorrect “strawman” GRN

## 3 Results

### 3.1 Simulation results for algorithm correctness and robustness

We show multiple simulations of the system with different field length *L* and goal RD peaks *N*. With a constant cell radius and cell-to-cell spacing, the field length is proportional to the number of cells in the simulation.

We picked our parameters to show a sampling of the system’s capabilities. The first three simulations use a field 200 cells long. In the first simulation, our goal is two peaks: i.e., a morphogen profile of LHLH. We start out (Table 1) with λ_RD_ = 7.2 × 10^−7^, which yields four peaks rather than the desired two. The top-level controller then directs seven iterations of counting, noting that there are too many peaks, and increasing λ_RD_ by 10% on each iteration, eventually giving the desired two peaks. The second simulation (Table 2) starts with the same initial conditions but has a goal of *N* = 5 rather than *N* = 2. This time, the controller goes through four iterations of decreasing λ_RD_, eventually creating the desired extra peak.

**TABLE 1:** Summary of simulations #1–3. Each row is one top-level-algorithm iteration. *N_peaks* is the current number of peaks found, and *N* is the target. *Action* describes the action of the top-level algorithm: whether it is increasing vs. decreasing λ_RD_.

Iteration	λ_RD_	n_peaks	N	Action
0	7.2 × 10^−7^	4	2	init
1	7.9 × 10^−7^	4	2	increase
2	9.6 × 10^−7^	4	2	increase
3	1.1 × 10^−6^	4	2	increase
4	1.2 × 10^−6^	3	2	increase
5	1.3 × 10^−6^	3	2	increase
6	1.4 × 10^−6^	3	2	increase
7	1.5 × 10^−6^	2	2	increase

**TABLE 2:** Summary of simulation #2.

Iteration	λ_RD_	n_peaks	N	Action
0	7.2 × 10^−7^	4	5	init
1	6.5 × 10^−7^	4	5	decrease
2	5.9 × 10^−7^	4	5	decrease
3	5.4 × 10^−6^	5	5	decrease

The third simulation (Table 3) shows an interesting quirk of some RD patterns. With the same 200-cell field, we started with a different initial λ_RD_. As noted above, a single field length *L* can often support various numbers of peaks at the same λ_RD_; it is a dynamic system with multiple stable points, and it is often difficult to know which stable point the system will travel to. We thus see iteration #1 setting λ_RD_ = 5.4 × 10^−7^, which could have given us five peaks but instead gave six. Iteration #3 hopped over five peaks directly to four. Eventually, though, the controller keeps adjusting λ_RD_ until it successfully reaches the goal. This ability to compensate for the unpredictability of RD patterns highlights a strength of our closed-loop feedback approach.

**TABLE 3:** Summary of simulation #3.

Iteration	λ_RD_	n_peaks	N	Action
0	4.9 × 10^−7^	6	5	init
1	5.4 × 10^−7^	6	5	increase
2	5.9 × 10^−7^	6	5	increase
3	6.5 × 10^−7^	4	5	increase
4	5.9 × 10^−7^	4	5	decrease
5	5.4 × 10^−7^	5	5	decrease

The fourth simulation (Table 4) uses a small 100-cell-long field. Unsurprisingly, the initial pattern had only two peaks rather than the four in Tables 1, 2. However, multiple iterations of decreasing λ_RD_ succeeded in reaching the target of four peaks.

**TABLE 4:** Summary of simulation #4. The field length is now only 100 cells, yielding an initial pattern of only two peaks. However, the algorithm reduces X_RD_ enough to reach the goal of four peaks.

Iteration	λ_RD_	n_peaks	N	Action
0	7.2 × 10^−7^	2	4	init
1	7.9 × 10^−7^	2	4	increase
2	9.6 × 10^−7^	2	4	increase
3	1.1 × 10^−6^	3	4	increase
4	1.2 × 10^−6^	3	4	increase
5	1.3 × 10^−6^	3	4	increase
6	1.4 × 10^−6^	3	4	increase
7	1.5 × 10^−6^	4	4	increase

The fifth simulation (Table 5) uses a long 300-cell field. It now starts with five peaks (rather than the four peaks of a 200-cell field). This time, multiple steps of increasing λ_RD_ enable it to reach the goal of three peaks.

**TABLE 5:** Summary of simulation #5; 300 cells. The longer field length results in more initial pattern repetitions, which is then countered by increasing λ_RD_.

Iteration	λ_RD_	n_peaks	N	Action
0	7.2 × 10^−7^	5	3	init
1	6.5 × 10^−7^	5	3	decrease
2	5.9 × 10^−7^	5	3	decrease
3	5.4 × 10^−6^	5	3	decrease
4	4.9 × 10^−6^	5	3	decrease
5	4.1 × 10^−6^	4	3	decrease
6	3.7 × 10^−6^	4	3	decrease
7	3.3 × 10^−6^	4	3	decrease
8	3.0 × 10^−6^	3	3	decrease

### 3.2 Simulation results from varying segment lengths

We have shown that varying λ_RD_ allows us to control the number of RD peaks we create. Once we have achieved the desired peak count, we can then vary λ_RD_ locally to further control pattern shape. Figure 3A shows a pattern with 100 cells that was targeted to a three-peak pattern. The peaks are originally at cells 20, 60 and 100 (blue graph). A subsequent simulation then slightly modifies the GRN so that any cell expressing *S0H* increases its λ_RD_ by 60% (Figure 3A, orange graph). Figure 3B is quite similar, except in this case we have modified the GRN so that any cell expressing either *S0H* or *S1L* increases its λ_RD_ by 60%. In each case, the appropriate segment(s) of the RD pattern increase in length at the expense of their immediate neighbors.

This capability is our digital equivalent of what Green (Green and Sharpe, 2015) calls “RD acting downstream of PI.” In his version, PI first lays down a coordinate system and then RD uses it to affect λ_RD_. In ours, our computation wave first lays down one or more coordinate systems and we can then stretch or shrink any subset of them almost arbitrarily.

## 4 Discussion

### 4.1 Choosing a feedback measure

Robustness is one of the great challenges in morphogenesis (Lander, 2007), and many strategies have evolved to achieve it. Many of them use negative feedback, which is an extremely common motif in nature (Alon, 2019). For example, mice use RD to reliably pattern fingers and toes (Raspopovic et al., 2014). To then prevent large embryos from having extra toes, mice use fibroblast growth factor (FGF), which affects embryo growth, to also increase λ_RD_. If FGF were the only factor affecting embryo growth, this strategy would be completely robust. However, in addition to the inherent unreliability in RD, any variations in embryo size from sources other than FGF are not controlled for, thus occasionally resulting in four- or six-toed mice.

In error-correcting control systems, you get what you measure. FGF concentration is serving as a proxy (albeit an imperfect one) for the field length *L*, and being used to control for the fact that increasing *L* would normally increase the number of RD pattern replications. In other words, the proxy for *L* is being fed *forward* to control λ_RD_. However, arguably the most appropriate goal is to preserve the number of toes–and the most reliable way to do that is not to measure *L* at all, but to directly measure the number of toes as a feedback mechanism.

Expansion-repression basically generates *A* at one end of the field at some rate *G*_A_, measures [*A*] at the other end, and increases (decreases) λ_RD_ when the distal [*A*] is too small(large). It is thus using the distal [*A*] as a proxy for *L*. If *L* doubles, then expansion-repression will double λ_RD_ (e.g., by quadrupling *D*_A_). The combination of doubling *L* and quadrupling *D*_A_ can easily be shown to exactly restore the original [*A*] profile. Since [*A*] affects λ_RD_, which then affects [*A*], this is indeed negative feedback.

If, instead of doubling *L*, we double *G*_A_, then the distal [*A*] will originally double. It is also easy to show that if we then double both *D*_A_ and *K*_D,A_, then the profile of [*A*] will be restored. However, in both these cases, if we restore the distal [*A*] by any other combination of changing *D*_A_ and *K*_D,A_, then [*A*] at the source will change, as well as the exact profile. The issue is that we are attempting to preserve an exact profile shape but using [*A*] at a single point to serve as a proxy for that profile.

Our top-level loop, measuring the number of peaks and altering λ_RD_ accordingly, is clearly a negative-feedback system. Specifically, our feedback variable is the number of pattern peaks, which is exactly the final variable most important to control. Thus, as long as our feedback system itself is operational, we will be immune from changes in other, non-feedback variables.

This is particularly important since RD patterns are not fully predictable. As shown by our simulation #3 and also noted in (Werner et al., 2015), while a pattern with characteristic length λ_RD_ will be stable on a field of length *L*, it may also be stable on any field of length longer than *L*. Similarly, increasing λ_RD_ such that (as noted above) a six-peak pattern is no longer viable may lead instead to a four-peak pattern, even though five peaks would be feasible. A field can thus often stably sustain a choice of multiple RD peak counts. Each choice has its own stability region around it, where unstable initializations will flow to that particular stable point. The stability regions are often difficult to predict.

The basic building blocks of biology are almost always noisy and difficult to predict (McAdams and Arkin, 1999). Building systems that nonetheless work reliably is thus often difficult, and our case is no exception. While our basic RD patterns can be difficult to use, the most effective feedback system–one where we close the loop by directly monitoring the variable we care most about–is quite effective. This is exactly the system we have built.

### 4.2 Using our digital breadcrumbs

While it is clear that morphogen gradients exist in nearly all complex organisms, it is less clear exactly how they are used. The gradients may be quite small (Warmflash et al., 2014; Tewary et al., 2017; Kaul et al., 2023). Furthermore, it is not known whether organisms read the morphogen concentration, its concentration gradient, a fold-change between neighboring cells, or something else (Simsek and Ozbudak, 2022). Since it is impractical to read a morphogen gradient with more than perhaps 10 levels (Simsek and Ozbudak, 2022), a simple gradient is clearly not enough to drive differentiation of a human body, which has over 3 × 10^13^ cells (Bianconi et al., 2013). A more plausible strategy is to divide and conquer, where the body first divides into (e.g.,) organs, which then form and differentiate independently from each other, each using its own smaller coordinate system. More complex organisms might create their form using even more levels of hierarchy. The task of forming a foot might involve subdivision into five smaller pieces, each with its own coordinate system, and then using a common “toe routine” to further develop each identical piece.

Our closed-loop RD system can do this easily, partitioning the partially-grown field of cells into smaller pieces, each with its own coordinate system that can be used by PI. We may further want different toes to take up different amounts of space, with a big toe typically being wider than the others, which would use our capability for unequal subdivision.

Our system of leaving digital breadcrumbs, via the Pre* signals, is a powerful means of letting each cell know which segment it is part of. It is somewhat reminiscent of Drosophila embryos, where maternal genes cause expression of the gap genes Kni, Hb, Kr and Gt; the embryo cells decode their position by decoding the combination of these four morphogens. It is believed (Petkova et al., 2019) that the embryo decodes these signals near optimally, though it is not exactly clear how. Since our Pre* signals are digital, it is quite easy to decode them; but it takes more morphogens than the Drosophila scheme (which uses analog values and thus requires fewer signals, but is less noise resistant).

### 4.3 Connecting our results to other work

Our work is complementary to Chhabra (Chhabra et al., 2019) in several ways. While we both use wavefronts as part of morphogenesis, the usage is somewhat different. Their wavefront is proposed as controlling differentiation; it is part and parcel of the cells’ fate decision. Ours, however, is more of a pure analysis wave, used simply to characterize an existing morphogen pattern as part of overall negative feedback.

Chhabra experimentally shows the occurrence of wavefronts, and that they are linked to cells’ fate decisions. They note that these decisions seem tied to the time between the WNT and NODAL wavefronts. However, they did not know the exact mechanism for this, nor how cellular fate decisions were remembered after the wave had passed. Our work could suggest a specific implementation mechanism for both of these unknowns; we detect and initiate computation upon detecting the WNT wavefront, and could easily measure the time between that and the subsequent NODAL wavefront.

Our work assumes that cells communicate by diffusion, with the diffused signals being potentially regenerated at each cell. However, Chhabra (Chhabra et al., 2019) shows that their signals are not regenerated, and are instead presumably traveling solely by diffusion. Unaided diffusion is an order-*N* ^2^ process, while their results show the waves traveling at constant speed. They thus suggest that some form of active transport may be involved. Others (Reddien, 2018; Bischof et al., 2020) have also seen a role for active transport of morphagens. If indeed this is the case, it would greatly simplify the GRN in our work–since the majority of our complexity is to implement effectively-unidirectional signaling, while active transport is often naturally unidirectional.

Another way to reduce the complexity of our GRN might be the imposition of an electric field. This would cause charged signaling ions to travel mostly unidirectionally, though diffusion still occurs–it would be interesting to see if this could simplify our model. Electrical waves have been observed in conjunction with potassium concentration in bacterial colonies (Prindle et al., 2015) and in Xenopus embryos (Vandenberg et al., 2011); our hypothesis may turn out to be a reason for those waves.

Tewary et al. (2017) describes a system where an RD system lays down one repetition of a pattern, which is then interpreted by PI to create the layers of the human gastrula. They show experimentally that increasing the size of the system can result in inadvertent repetitions of the RD pattern. Since larger human embryos do not have multiple gastrulas, they conclude that a higher-level system is probably preventing that. Our work fits nicely with this view; we have proposed a candidate high-level system.

### 4.4 Limitations

A limitation of our approach is that we have posed our problem as starting with an existing field of cells and subdividing it. However, most embryos are growing at the same time as cells are differentiating, and cells are migrating as well. Our simpler case is not uncommon, though. Mammalian and avian blastoderms can divide in two to create identical twins; each of the two new embryos then reforms itself, differentiating anew to alter each of the existing embryos. Planarian morphallaxis is another interesting case; when an adult planarian is cut into fragments, each fragment can regrow into a full new worm. However, since a fragment may be missing a mouth or indeed an entire digestive system, the fragment cannot increase its mass until it has the capability of eating. It thus undergoes morphallaxis (Reddien, 2018; Reddien, 2021), where the fragment reforms itself into a fully formed but small-scale planarian, and then eats and grows.

We have similarly discussed the problem as occurring in discrete steps; the top-level controller measures a morphological property via a computation wave, then alters parameters, waits for them to take effect, and repeats until it converges to the target. Others (Friston et al., 2015; Friston, 2019) have suggested that the process is more asynchronous and continuous; that each cell continuously monitors if its expectations for the cells in its neighborhood match what it currently senses, and migrates or differentiates so as to bring the two into accord. The large collection of independent agents would march towards convergence by a free-energy-minimization process. We do not know which hypothesis is correct; it could be that a combination of both happens, and computation waves are used to calculate their form of free energy. In the case of RD patterns, however, they tend to have to converge to a fixed pattern before their shape makes any sense at all, which would seem to fit our approach.

The use of discrete signals in negative-feedback controllers in nature is not uncommon. Bacterial chemotaxis uses an integral-based feedback system (Alon, 2019) to decide how often to tumble (i.e., to try a new direction in a search for food). A receptor can be methylated in any of five locations; more methylation implies a larger frequency of tumbles. The controller, like ours, uses a discrete variable with a small number of legal values to serve its purpose.

This work is purely *in silico*; we have yet no evidence that this particular negative-feedback system exists in nature. However, it seems fairly clear that *some* sort of negative-feedback system must exist in morphogenesis. The ability of a mammalian embryo to successfully recover from disturbances as varied as being split in two (e.g., for identical twins) and transient mRNA interference to the early embryo (Vandenberg et al., 2012) would be difficult to explain otherwise. Regardless of whether evolution found exactly this scheme, it can now be used in synthetic biology approaches to engineer novel patterning systems (Tiwary et al., 2015; Velazquez et al., 2018; Santorelli et al., 2019; Silva-Dias and Lopez-Castillo, 2022).

As noted in the introduction, measuring waves is technically challenging. While waves have been observed the lab, confirming our hypothesis would be much easier if future work creates transgenics with fluorescent reporters that allow readouts of the morphological computation in the living state.

## 5 Conclusion and future work

We have demonstrated a closed-loop negative-feedback machine to control morphogenesis, as a contribution to the efforts to understand morphogenesis as a target-directed process (Levin et al., 2023). Its goal is to lay down *N* copies (for a reasonably arbitrary *N*) of a simple RD pattern; e.g., to be used as repeats of a coordinate system. It achieves this goal with a closed-loop negative-feedback controller that

• employs waves to count morphogen peaks, and thus count the current number of pattern repetitions. The waves work by taking advantage of existing asymmetry.
• compares the current number of peaks to its goal *N.*
• adjusts the RD pattern-length parameter λ_RD_ so as to move the pattern towards the goal.

By combining these concepts, we have

• created multiple-peak RD patterns more reliably than previous work
• as such, laid down multiple gradients for PI acting downstream of RD
• controllably made a subset of the pattern segments larger or smaller than the others, so as to subdivide a field into unequal subfields in a repeatable manner.

The circuit described here enables flexible actions under a range of circumstances to reach a specific large-scale goal state which belongs to the collective and not the individuals (Levin, 2019; Levin, 2022). Such capacity has been proposed as a definition of intelligence (Fields and Levin, 2022), for the field of basal cognition (Lyon, 2006; Lyon, 2015; Sole et al., 2016; Urrios et al., 2016; Macia et al., 2017). Thus, the above circuit and analysis not only supports a way of viewing morphogenetic processes as a set of specific computational tasks, but also suggests a synthetic biology architecture for incorporating a simple kind of intelligence into novel biological constructs (Doursat et al., 2013; Doursat and Sanchez, 2014; Kamm and Bashir, 2014; Kamm et al., 2018; Davies and Glykofrydis, 2020; Glykofrydis et al., 2021; Davies and Levin, 2022). Future work will investigate the presence of these dynamics *in vivo*, as well as use the insights revealed by this modeling process to create novel patterning systems for synthetic biorobotics, regenerative medicine, and tissue bioengineering.

## Data Availability

The raw data supporting the conclusion of this article will be made available by the authors, without undue reservation.
